# Periconceptional Folic Acid Supplementation and the Risk of Spontaneous Abortion among Women Who Prepared to Conceive: Impact of Supplementation Initiation Timing

**DOI:** 10.3390/nu12082264

**Published:** 2020-07-29

**Authors:** Yan-Yan Mao, Liu Yang, Min Li, Jun Liu, Qian-Xi Zhu, Yang He, Wei-Jin Zhou

**Affiliations:** 1 NHC Key Laboratory of Reproduction Regulation, Shanghai Institute of Planned Parenthood Research, Fudan University, Shanghai 200237, China; maoyanyan@sippr.org.cn (Y.-Y.M.); liminnl@sina.com (M.L.); qianxizhu@hotmail.com (Q.-X.Z.); zw0822@sina.com (W.-J.Z.); 2 NHC Key Laboratory of Birth Defects and Reproductive Health, Chongqing Population and Family Planning Science and Technology Research Institute, Chongqing 400020, China; lily882@tom.com (L.Y.); lj790717@126.com (J.L.)

**Keywords:** folic acid, supplementation, periconceptional, initial time, spontaneous abortion

## Abstract

It is unclear whether periconceptional folic acid (FA) supplementation decreases the risk of spontaneous abortion (SA). The impact of supplementation initiation timing has not been ascertained. This cohort study aimed to investigate the association between maternal periconceptional FA supplementation and risk of SA, with due consideration of the supplementation initiation timing. Through the National Free Pre-conception Health Examination Project (NFPHEP), we identified 65,643 pregnancies on FA supplementation in Chongqing, China between 2010 and 2015. After adjusting for covariates, maternal periconceptional FA supplementation was associated with a lower risk of SA (adjusted risk ratio [aRR]: 0.52; 95% confidence interval [CI]: 0.48–0.56). Pregnant women with FA supplementation initiated at least 3 months before conception had a 10% lower risk of SA (aRR: 0.46; 95% CI: 0.42–0.50) than those with FA supplementation initiated 1–2 months before conception (aRR: 0.56; 95% CI: 0.50–0.62) or after conception (aRR: 0.56; 95% CI: 0.51–0.61). These associations might not thoroughly account for FA supplementation, and to some extent our findings confirm the role of the utilization of healthcare in preventing SAs. Women who initiated healthcare, including taking FA earlier during the periconceptional period, could have a lower risk of SA.

## 1. Introduction

Spontaneous abortion (SA), or miscarriage, is defined in China as fetal loss before 28 gestational weeks [1,2,3]. SA occurs in at least 15% pregnancies and is strongly associated with infertility as the severity of reproductive failure is a common cause and risk factor for SA [2,3,4]. Furthermore, women who experience SA have a higher risk of developing psychiatric conditions, such as depression or anxiety, in the first year after SA [5].

Nearly 50% of SAs result from chromosomal aneuploidy [6]. Accordingly, periconceptional supplementation with folic acid (FA), a synthetic form of folate that is essential for one-carbon metabolism and DNA synthesis, repair, and methylation [7], may reduce the risk of SA. Maternal FA supplementation before or during pregnancy has been established to be beneficial for the prevention of neural tube defects (NTDs) and other birth defects [8,9,10] that confer a risk of fetal chromosomal abnormalities [11]. However, there is no consensus regarding the association between FA supplementation and SA [11,12,13,14,15]. Moreover, the benefit of FA supplementation in reducing the risk of SA may depend on the time at which supplementation is initiated. A previous study reported that women who started supplementation prior to conception were more likely to achieve optimal folate levels necessary to prevent NTDs than women who started supplementation after conception [16]. Furthermore, pre-conception FA supplementation has been reported to be associated with a lower risk of preterm delivery than supplementation initiated after conception [17]. However, there is a lack of evidence on the association between the timing of FA supplementation initiation and its effect on the prevention of SA. Thus, this cohort study aimed to investigate the association between maternal periconceptional FA supplementation and SA risk and examined whether the timing of supplementation initiation affected the SA risk.

## 2. Materials and Methods

### 2.1. Study Population

Participants originally comprised pregnant women on FA supplementation in Chongqing, China between 2010 and 2016, who were identified through the National Free Pre-conception Health Examination Project (NFPHEP). In total, 96,722 women with 97,154 pregnancies were identified. Among these pregnancies, we excluded 230 pregnancies in which the women did not adhere to guidance on FA supplementation, 186 ectopic pregnancies, 29,299 pregnancies that were lost to follow-up, and 1796 pregnancies recruited in 2016 due to the high rate lost to follow-up (>80%) in 2016 (Supplementary Table S1). Thus, 65,497 women with 65,643 pregnancies were included in the final analyses between 2010–2015 (Supplementary Figure S1).

### 2.2. National Free Pre-Conception Health Examination Project

The NFPHEP was launched to provide free pre-conception health examinations and counseling services to couples who planned to conceive [18]. All women participating in the NFPHEP were interviewed in person by local trained physicians using a structured questionnaire. Demographic characteristics and history of pregnancies were assessed. The height and weight were measured by the physicians using calibrated instruments. After the interview and physical examination, the participants were followed-up through telephone calls every 3 months to check for the status of their conception/pregnancy.

Once pregnancy was confirmed, the last menstrual period, FA supplementation status, and tobacco and alcohol consumption before or during early pregnancy of the participant and spouse were documented. In the following interviews, the pregnancy outcomes were reported by the participants or retrieved from the medical records in the hospitals where they delivered. All the information collected at recruitment or during follow-up was filed in the NFPHEP records.

### 2.3. Exposure, Outcomes, and Covariates

Information on exposure, outcomes, and covariates was extracted from the NFPHEP records. The exposure variable was maternal periconceptional FA supplementation, which was determined after confirmation of conception. We obtained information regarding whether the women took FA supplements during the periconceptional period, when they began to take the supplements, and how regularly they took the supplements. The regular supplementation was hypothesized that women were compliant to the recommendation, although the information on participants’ daily dose of folic acid in supplements was unavailable in the NFPHEP records. Women planning to conceive are advised to consume 0.4 mg folic acid tablets daily in the periconceptional period (from 3 months before conception until 12 weeks thereafter) in China [19]. The pregnant women who adopted FA supplementation during the periconceptional period were categorized as the FA group, and those who did not use FA supplements as the no-FA group. The FA group was further divided into three subgroups based on the initiation of supplementation: Starting 3 months prior to conception, 1–2 months prior to conception, or after conception.

The outcome measure was SA, which was defined according to clinical classification of obstetrics and gynecology in China as the loss of pregnancy before 28 complete gestational weeks [20]. The non-SA outcomes included induced abortions, stillbirth, and liveborn deliveries. Stillbirth was the death or loss of a baby before or during delivery after 20 weeks of pregnancy [20]. The SAs and stillbirths in our study were reported by the women and confirmed by the local obstetricians and gynecologists.

The covariates were maternal age at recruitment (20–24, 25–29, 30–34, and ≥35 years), ethnicity (Han and others), education (primary school or below, junior high school, senior high school, college/university graduate or above), employment (farmer, worker/waiter, merchant, housewife, teacher/civil servant/staff, and others), household registration (urban and rural), calendar year, and recruitment area (urban core area, new urban district, northeast ecological conservation area, and southeast ecological protection area of Chongqing). Additional covariates included maternal passive smoking before pregnancy; husbands’ smoking habits during early pregnancies; maternal pre-pregnancy body mass index ((BMI); <18.5, 18.5–23.9, 24–27.9, and ≥28 kg/m^2^); history of complicated pregnancies (preterm delivery, stillbirth, and spontaneous/induced abortion); birth defect or history of a birth defect in a previous delivery; and chronic diseases, including thyroid disorders, hypertension, diabetes, epilepsy, and mental illness before pregnancy. Pregnant women with missing information for one of the co-variates were classified into one group.

### 2.4. Statistical Analysis

All statistical analyses were conducted in Stata 15.0 (Stata Corp, College Station, TX, USA). Binomial regression was used to estimate the risk ratio (RR) and 95% confidence interval (CI) for SA using the no-FA group as the reference group. Moreover, the RRs for the three subgroups of supplementation initiation timing were also estimated using the no-FA group as the reference group. The differences between subgroups were examined using a Wald *chi*-square test.

Both crude and adjusted RRs of SA were calculated. The adjusted analyses controlled for maternal age, ethnicity, education, employment, household registration, calendar year, and area of recruitment at first. The results were further controlled for factors including maternal BMI, passive smoking before the pregnancy, parity, previous complicated pregnancies, chronic diseases, birth defects or a history of a birth defect in a previous delivery, and husbands’ smoking habits during early pregnancy. We estimated the risk of SA according to the maternal characteristics and with respect to how regularly the women took the supplements (compliance with FA supplementation). To account for the correlation of pregnancies from the same women when estimating standard errors, we used the cluster option in Stata.

In the sensitivity analyses, we investigated the influence of the following factors on the association between FA supplementation and SA: (1) Maternal infection including reproductive tract infection (RTI), cytomegalovirus (CMV), and toxoplasma (TOX) infection before pregnancy; (2) the present pregnancies ending with induced abortion/stillbirth; (3) missing information on covariates in the study group and subgroups of FA supplementation; and (4) loss to follow-up. Furthermore, to assess the robustness of the reported results to residual confounding, we calculated the E-values. The calculation of the E-values is a type of sensitivity analysis used to estimate the potential impact of an unmeasured confounding factor on the RR estimates. The E-value is defined as the minimum strength of association on the RR scale that an unmeasured confounder would need to have with both the exposure (FA supplementation) and the outcome (SA) to fully explain a specific exposure–outcome association [21].

### 2.5. Ethics Approval and Consent to Participate

This cohort study was a part of the project funded by the Chongqing Science and Technology Bureau (grant number cstc2017shmsA130102) and approved by the institutional review board of Chongqing Population and Family Planning Science and Technology Research Institute in April 2017. The NFPHEP was approved by the institutional review board of the Chinese Association of Maternal and Child Health Studies. Written informed consent was obtained from all participants before the survey.

## 3. Results

### 3.1. Maternal Characteristics

In this cohort, the participants were mostly recruited 2 months before pregnancy onset and were generally followed up 3 months after conceiving for the first time. In total, 50,404 (76.79%) pregnant women had periconceptional FA supplementation, while 15,239 (23.21%) pregnant women had no supplementation. In the FA group, 42.02% started supplementation at least 3 months prior to conception, 23.47% started 1–2 months prior to conception, and 34.51% started after conception. The characteristics of these women are summarized in Supplementary Table S2. The median maternal age was 25 years (interquartile range: 22–27 years). Pregnant women who were older urban residents and had a higher educational level were more likely to start FA supplementation earlier. Pregnant women who were passive smokers, previously had complicated pregnancies, chronic diseases, and/or birth defects or history of a birth defect in a previous delivery were also more likely to start FA supplementation earlier. However, pregnant women whose husbands smoked during early pregnancy were less likely to receive FA supplementation. Pregnant women who started FA supplementation earlier tended to present with more regular FA supplementation.

### 3.2. The Association between Maternal FA Supplementation during Periconceptional Period and the Risk of SA

**Figure 1:**
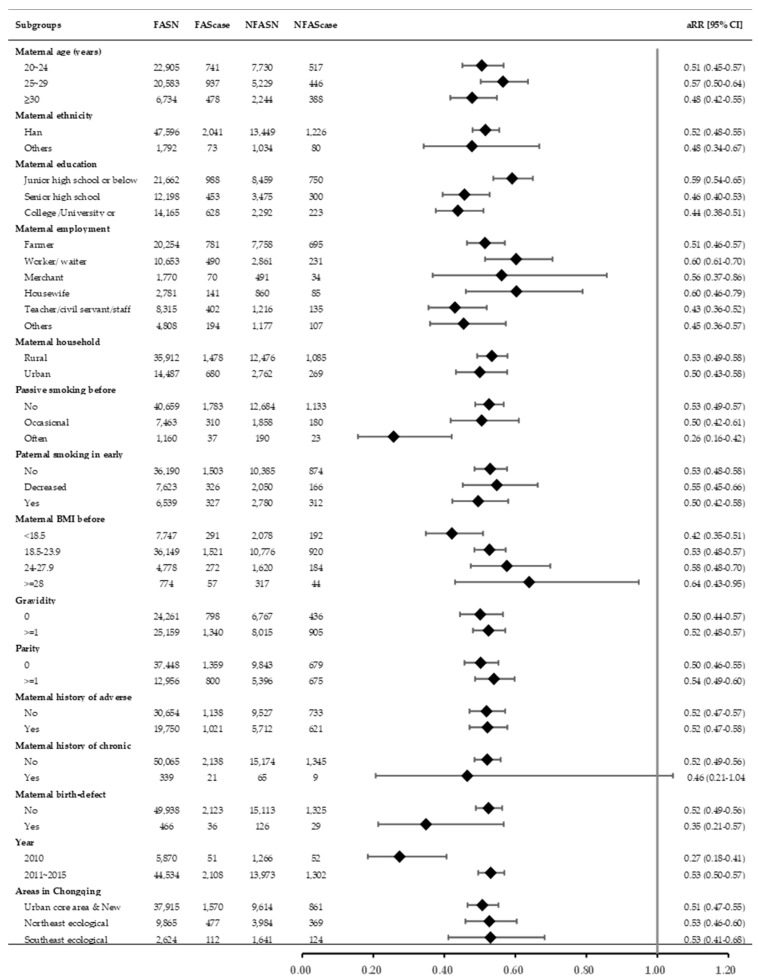
Stratification analyses on the association between folic acid (FA) supplementation and the risk of spontaneous abortion according to maternal characteristics. All risk ratios adjusted for maternal characteristics other than the stratified characteristic. FASN and FAScase: Pregnancy and spontaneous abortion (SA) number, respectively, in the FA group. NFASN and NFAScase: Pregnancy and SA number, respectively, in the no-FA group.

Among the pregnancies included in the final analysis, 3513 (5.35%) ended with SA. The proportion of SA was 3.78%, 4.57%, 4.70%, and 8.89% for the 3 months prior to conception, 1–2 months prior to conception, after conception FA groups and the no-FA group, respectively (Table 1). Compared to pregnant women in the no-FA group, women in the FA group had a lower risk of SA (crude risk ratio (cRR): 0.48; 95% CI: 0.45–0.51) and the association did not markedly change (adjusted risk ratio (aRR): 0.52; 95% CI: 0.48–0.56) after adjustment for covariates (Table 2). In the analyses stratified by maternal characteristics, the associations also did not markedly change, although the corresponding aRRs between the subgroups were different according to maternal education, passive smoking, pre-pregnancy BMI, and recruitment year (Figure 1).

**Figure 2:**
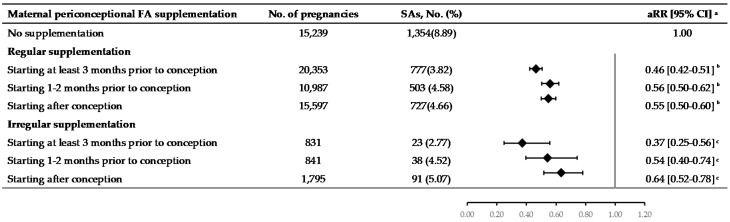
Subgroup analysis on the association between maternal periconceptional folic acid (FA) supplementation and the risk of spontaneous abortion (SA) according to compliance to supplementation and the initiation timing of supplementation. ^a^ The RRs were adjusted for maternal age, ethnicity, education, employment, household, registration, year and area of recruitment, BMI, passive smoking before pregnancy, parity, history of complicated pregnancies, chronic diseases, birth defects or history of a birth defect in a previous delivery, and husbands’ smoking in early pregnancy. ^b^ The aRR for FAS initiated 3 months or more prior to conception and risk of SA was significantly lower than that for FAS initiated 1–2 months prior to conception ($\chi^{2}$ = 11.29, *p* = 0.0011) or after conception ($\chi^{2}$ = 10.59, *p* = 0.0011). ^c^ The aRR for FAS initiated 3 months or more prior to conception was significantly lower than initiated after conception ($\chi^{2}$ = 5.53, *p* = 0.0187). aRR, adjusted risk ratio; CI, confidence interval.

Further analysis on the association between FA supplementation and the risk of SA according to the initiation time of FA supplementation showed that pregnant women who started the supplementation at least 3 months prior to conception had a 10% lower risk (aRR: 0.46; 95% CI: 0.42–0.50) than either starting 1–2 months prior to conception (aRR: 0.56; 95% CI: 0.50–0.62; χ^2^ = 12.56) or after conception (aRR: 0.56; 95% CI: 0.51–0.61; χ^2^ = 14.61) (Table 2). In the subgroup analysis based on compliance to FA supplementation, pregnant women who started FA supplementation at least 3 months prior to conception continued to have a lower risk for SA regardless of how regularly they took supplementation (regular: aRR = 0.46; 95% CI: 0.42–0.51; irregular: aRR = 0.37; 95% CI: 0.25–0.56; Figure 2).

### 3.3. Sensitivity Analysis

**Figure 3:**
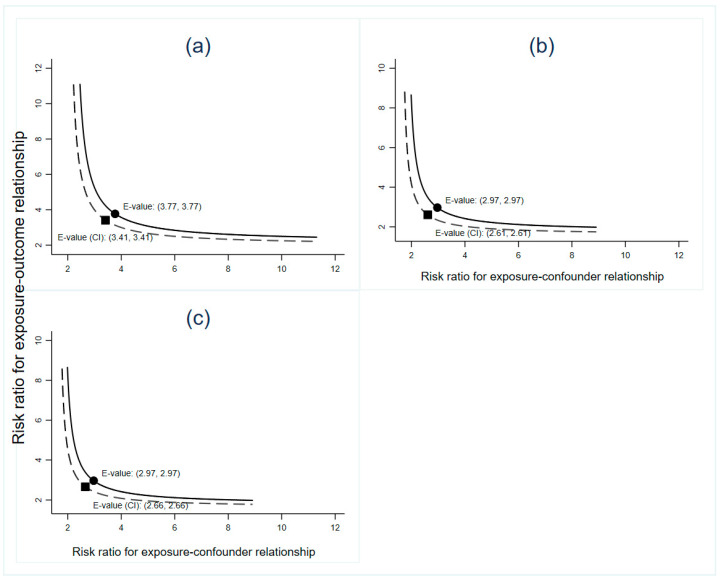
Value of the joint minimum strength of association on the risk ratio scale that an unmeasured confounder would be required to have with FA supplementation (the exposure), and spontaneous abortion (the outcome) to fully explain their observed RR according to initiation time of the supplementation: (**a**) Initiated 3 or more months prior to conception and the observed RR of 0.46 (95% CI: 0.42–0.50); (**b**) initiated 1–2 months prior to conception and the observed RR of 0.56 (95% CI: 0.50–0.62); (**c**) initiated after conception and the observed RR of 0.56 (95% CI: 0.51–0.61). FA, folic acid; CI, confidence interval; RR, risk ratio.

Furthermore, the associations did not significantly change even after adjusting for maternal RTI, CMV, or TOX infection before pregnancy, excluding pregnancies that ended with induced abortion/stillbirth, or excluding pregnant women without information for any covariates (Supplementary Table S3). The sensitivity analyses evaluated the robustness of the associations that we observed to unmeasured confounding. The observed RRs of 0.46, 0.56, and 0.56 for the outcome of SA in relation to FA supplementation initiated at least 3 months prior to conception, 1–2 months prior to conception, and after conception, respectively (Table 2), estimated that an unmeasured confounder was associated with both FA supplementation and SA by RRs of at least 3.77, 2.97, and 2.97 for FA supplementation initiated at least 3 months prior to conception, 1–2 months prior to conception, and after conception, respectively (Figure 3), beyond the measured confounders, but not by the weaker confounding factor. The corresponding CIs may be moved to include the null by an unmeasured confounder associated with both exposure (FA supplementation initiated at least 3 months prior to conception, 1–2 months prior to conception, and after conception) and outcome (SA) by RRs of at least 3.41, 2.61, and 2.66, respectively (Figure 3).

## 4. Discussion

The association of FA supplementation during the periconceptional period with the risk of SA remains unclear. In this population-based cohort study of 65,643 pregnancies among women who planned to conceive in Chongqing, China, 5.35% of the pregnancies ended in SA. FA supplementation before or during pregnancy decreased the risk of SA by 49%, and the decrease in risk was greater when supplementation started at least 3 months prior to conception. The association remained in the subgroup analysis pertaining to the participant’s compliance to supplementation and maternal characteristics.

Several studies have investigated the association between periconceptional FA supplementation and the risk of SA, but the results have been inconclusive. The MRC Vitamin Study, an earlier multi-center randomized controlled trial designed primarily for reducing the recurrence of NTDs across European countries, Canada, and Australia [22], observed a minor increase in risk for SA in pregnancies with FA supplementation during the periconceptional period. In the same period, FA supplementation from another randomized trial conducted in Hungary for the reduction of the first occurrence of NTDs was associated with a 16% increase in the risk of SA [12]. However, later studies in China as well as in an orofacial cleft prevention trial from Brazil found no such association between FA supplementation and SA [13,14]. This observational study found a lower risk for SA among the pregnant women with FA supplementation before or during pregnancy. This finding was consistent with that reported by He et al. (2016) [15], who reported that FA supplementation before or during pregnancy reduced the risk for SA by almost 40%.

The varied findings mentioned above might result from different study designs or difference in populations. Most previous studies investigated the usefulness of FA supplementation for the prevention of birth defects [12,13,14,22]. The presence or absence of an NTD was usually confirmed after 20 weeks of gestation period, but the age of gestation of pregnancy loss to define SA varied between the studies, ranging from 20 to 24 complete gestational weeks [3,23]. In China, SA is clinically defined as pregnancy loss before 28 complete gestational weeks. The incidence of SA might be lower in China (4.36% between 1988 and 1997) [24] while Chongqing in southwest of China might have a higher incidence than the whole country (5.35% VS. 2.53% based on NFPHEP) [25]. In addition, pregnancies identified in the present study were from the general population of pregnant women who planned to conceive rather than the high-risk or primiparous population.

The initiation time of FA supplementation has been theorized to impact the association between FA supplementation and SA. Internationally, many governments recommend women take ≥0.4 mg of folic acid daily starting at least 4 weeks before pregnancy, according to the guideline by World Health Organization (2007). Meanwhile, more than 80 counties adopt a mandatory FA fortification policy. Differently, despite no food fortification in China, a national recommendation has been released since 2009 that women who plan a pregnancy are advised to take 0.4 mg FA tablets daily from 3 months before conception until 12 weeks thereafter. In this study, we found that the benefit of FA supplementation for reducing the risk of SA was more profound in pregnant women who started the supplementation at least 3 months before conception. Further, regular FA supplementation was more common among the women who started 3 months or more prior to conception than in those who started 1–2 months before and those who started after conception. These findings indicate better compliance and a more optimal folate concentration against chromosome abnormalities and birth defects in the women who started FA supplementation earlier [26,27]. Likewise, women who took FA earlier prepared for pregnancy earlier and thus may be in a healthier condition [28].

Our findings might not be completely attributed to the effects of FA supplementation because the supplementation has been associated with an array of maternal and paternal factors and may be a proxy for all these factors. Other determinants of SA such as maternal and paternal age, educational level, fertility treatment, experiencing a complicated pregnancy, unplanned pregnancy, alcohol consumption, and smoking have also well predicted the use of FA supplementation in periconceptional period [29,30,31,32]. Maternal smoking and BMI may also modify the association between FA supplementation and SA [3]. Recently, one systematic review showed that second-hand smoking exposure during pregnancy is generally associated with lower folate/vitamin B12 levels [33]. The protective effect from FA supplementation on SA may therefore be decreased by smoking exposure or obesity [34]. However, the lower risk of SA in the FA group still remained after adjusting for or stratifying by these maternal characteristics.

One of the possible mechanisms linking FA supplementation to the reduced risk of SA may be attributable to the role of FA in meiosis. Almost 50% of SAs result from fetal chromosomal abnormalities, of which a high proportion is caused by maternal or paternal meiosis. FA deficiency delayed the onset of meiosis in male mice and increased DNA damage in spermatocytes [35]. Moreover, FA deficiency has been reported to possibly increase the risk for meiosis II nondisjunction errors among women aged older than 35 years [36].

This cohort study was conducted with prospectively recorded exposure data together with the available confounders. Its large sample size of 65,643 pregnancies enabled us to control for the influencing confounders on the association between FA supplementation and SA. However, our findings should be interpreted with caution and generalized carefully owing to several limitations. Firstly, our study population was pregnant women who planned to conceive and took part in NFPHEP to have the preconception healthcare. Those who did not plan to conceive were not recorded in this project. Secondly, the pregnant women would be more likely to have FA supplementation if the gestation was prolonged. However, we categorized the pregnancies according to time of the first follow-up during pregnancy and found no association between FA supplementation and the time of follow-up (Supplementary Table S4). Thirdly, the covariates could not be completely balanced in the study, and the unmeasured covariates may have biased the estimation. Taking dietary folate, for example, which was varied in seasons and regions, would have biased our estimate. In recent years, the variation is being reduced due to FA supplementation program since 2009 and the high access to green vegetables and fresh fruits in winter or spring. Besides, the adjustment for the maternal demographic and social characteristics, which were related to dietary folate intake, may also partly control the bias. We estimated the E-value to assess the robustness of association to potential unmeasured confounding. The values were relatively large in our study as most of the known covariates had an RR of less than 3.00; the residual confounding could explain the observed association whether there exists an unmeasured covariate that has an RR association at least as large as the estimated value with both FA supplementation and with SA. Despite such an unmeasured or unknown covariate possibly existing, the possibility is small. Furthermore, pregnant women with chronic diseases such as hypertension had a higher risk of SA than pregnant women without these diseases [37]. Our analysis, adjusted for chronic diseases and other maternal characteristics, showed that FA supplementation was still associated with a lower risk of SA. Thirdly, 29,293 (30.22%) of the 96,924 pregnancies were lost to follow-up (31.5% in the FA group and 25% in the no-FA group). We analyzed the association between FA supplementation and the risk of SA in a subpopulation that was recruited in 2010 and of which only 232 (3.15%) pregnant women were lost to follow-up. In this subpopulation, there was a consistent association between FA supplementation and SA (Supplementary Table S5). Fourthly, 2168 (3.30%) of the pregnancies ended in induced abortion/stillbirth. Although we were unable to observe the natural outcome for most of these pregnancies, 96.0% of the induced abortions/stillbirths were reported to be due to fetal abnormalities. Additionally, the association between FA supplementation and SA did not change after excluding the induced abortions/stillbirths in the sensitivity analysis (Supplementary Table S3) and the pregnant women with FA supplementation had a 70% lower risk of stillbirth or induced abortion upon further analysis (Supplementary Table S6).

## 5. Conclusions

Maternal periconceptional FA supplementation is associated with a reduced risk of SA independent of known confounding factors. This reduction in the risk was more profound in earlier initiation of FA supplementation, that is, at least 3 months prior to conception. These findings provide new evidence concerning maternal FA supplementation in pre-conception and antenatal care.

## Figures and Tables

**Table 1:** Pregnancy outcomes according to maternal periconceptional folic acid (FA) supplementation.

Pregnancy Outcomes	Starting at Least 3 Months before Pregnancy (*N* = 21,184), No. (%)	Starting 1–2 Months before Pregnancy (*N* = 11,828), No. (%)	Starting after Conception (*N* = 17,392), No. (%)	No FA Supplementation (*N* = 15,239), No. (%)	Total (*N* = 65,643), No. (%)
Live birth	19,988 (94.35)	11,046 (93.39)	16,122 (92.70)	12,806 (84.03)	59,962 (91.35)
Spontaneous abortion	800 (3.78)	541 (4.57)	818 (4.70)	1354 (8.89)	3513 (5.35)
Induced abortion/Stillbirth	396 (1.87)	241 (2.04)	452 (2.60)	1079 (7.08)	2168 (3.30)

**Table 2:** Risk ratios and 95 CIs for spontaneous abortion (SA) according to maternal periconceptional folic acid (FA) supplementation.

Maternal Periconceptional FA Supplementation	No. of Pregnancies	SAs, No. (%)	cRR [95% CI]	aRR [95% CI] ^a^
No supplementation	15,239	1354 (8.89)	1.00	1.00
Having supplementation	50,404	2159 (4.28)	0.48 [0.45–0.51]	0.52 [0.48–0.56]
Starting at least 3 months prior to conception	21,184	800 (3.78)	0.43 [0.39–0.46]	0.46 [0.42–0.50]
Starting 1–2 months prior to conception	11,828	541 (4.57)	0.51 [0.47–0.57]	0.56 [0.50–0.62]
Starting after conception	17,392	818 (4.70)	0.53 [0.49–0.58]	0.56 [0.51–0.61]

**^a^** aRR adjusted for maternal age, ethnicity, education, employment, household registration, year and area of recruitment, BMI, passive smoking before pregnancy, parity, history of complicated pregnancies, chronic diseases, birth defects or history of a birth defect in a previous delivery, and husbands’ smoking in early pregnancy. The aRR for FA supplementation initiated at least 3 months prior to conception was significantly lower than that for FA supplementation initiated 1–2 months prior to conception ($\chi^{2}$ = 12.56, *p* = 0.0004) or after conception ($\chi^{2}$ = 14.61, *p* = 0.0001). cRR, crude risk ratio; aRR, adjusted risk ratio; CI, confidence interval.

## Supplementary Materials

The following are available online at https://www.mdpi.com/2072-6643/12/8/2264/s1, Figure S1: Flowchart of the pregnancies included in the study, Table S1: Characteristics of pregnant women according to maternal response status between 2010 and 2016, Table S2: Characteristics of pregnant women according to periconceptional folic acid (FA) supplementation, Table S3: Risk ratios and 95 CIs for spontaneous abortion according to maternal periconceptional folic acid (FA) supplementation in sensitivity analysis, Table S4: The association between maternal periconceptional folic acid (FA) supplementation and the follow-up time in gestation, Table S5: Risk ratios and 95% CIs for spontaneous abortion (SA) according to maternal periconceptional folic acid (FA) supplementation in a subpopulation recruited in 2010 (*N* = 7136), Table S6: Risk ratios and 95% CIs for pregnancies ending with stillbirth or induced abortion due to fetal abnormalities according to maternal periconceptional folic acid (FA).
