# Supplementary material for: Periconceptional Folic Acid Supplementation and the Risk of Spontaneous Abortion among Women Who Prepared to Conceive: Impact of Supplementation Initiation Timing
Source: Nutrients. 2020 Jul 29;12(8):2264. doi: 10.3390/nu12082264 (PMC7469034; doi:10.3390/nu12082264)
Supplement: Supplementary file 1 [file nutrients-12-02264-s001.pdf]

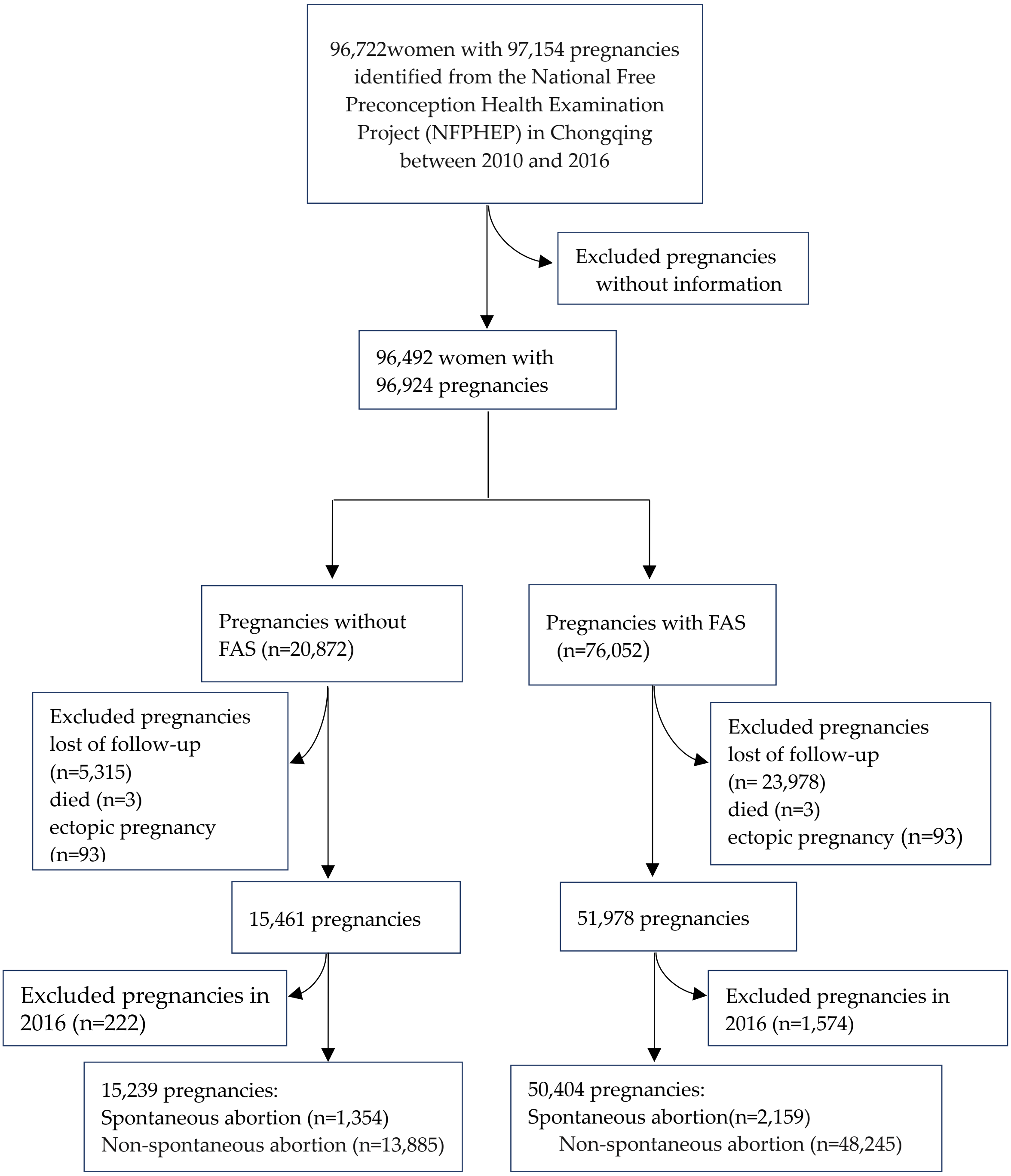

Figure S1. Flowchart of the pregnancies included in the study.

Table S1. Characteristics of pregnant women according to maternal response status between 2010 and 2016.

Maternal characteristics	Response				Total	
	Yes		No			
	(N=67,439)		(N=29,485)		(N=96,924)	
	n	%	n	%	n	%
Maternal periconceptional FA supplementation						
No supplementation	15,461	22.93	5,411	18.35	20,872	22.93
Starting after conception	17,926	26.58	7,260	24.62	25,186	26.58
Starting 1-2 months prior to conception	12,258	18.18	6,069	20.58	18,327	18.18
Starting at least 3 months prior to conception	21,794	32.32	10,745	36.44	32,539	32.32
Maternal age (years)						
20–24	31,318	46.44	13,210	44.80	44,528	46.44
25–29	26,516	39.32	11,473	38.91	37,989	39.32
30–34	6,666	9.88	3,259	11.05	9,925	9.88
≥35	2,721	4.03	1,432	4.86	4,153	4.03
Missing	218	0.32	111	0.38	329	0.32
Maternal ethnicity						
Han	62,740	93.03	27,338	92.72	90,078	93.03
Others	2,926	4.34	1,899	6.44	4,825	4.34
Missing	1,773	2.63	248	0.84	2,021	2.63
Maternal education						
Primary school or below	2,771	4.11	1,224	4.15	3,995	4.11
Junior high school	28,124	41.70	12,289	41.68	40,413	41.70
Senior high school	16,075	23.84	6,932	23.51	23,007	23.84
College /University or above	17,058	25.29	8,261	28.02	25,319	25.29
Missing	3,411	5.06	779	2.64	4,190	5.06
Maternal employment						
Farmer	28,618	42.44	12,845	43.56	41,463	42.78
Worker	6,646	9.85	2,752	9.33	9,398	9.70

waiter	7,253	10.75	2,948	10.00	10,201	10.52
Merchant	2,332	3.46	1,063	3.61	3,395	3.50
Housewife	3,767	5.59	1,458	4.94	5,225	5.39
Teacher/civil servant/staff	9,897	14.68	4,759	16.14	14,656	15.12
Others	6,222	9.23	3,171	10.75	9,393	9.69
Missing	2,704	4.01	489	1.66	3,193	3.29
Maternal household registration						
Rural	49,631	73.59	21,489	72.88	71,120	73.59
Urban	17,802	26.40	7,995	27.12	25,797	26.40
Missing	6	0.01	1	0.00	7	0.01
Maternal BMI before pregnancy (kg/m^2)						
<18.5	10,029	14.87	4,350	14.75	14,379	14.87
18.5–23.9	48,180	71.44	20,957	71.08	69,137	71.44
24–27.9	6,662	9.88	3,235	10.97	9,897	9.88
≥28	1,155	1.71	556	1.89	1,711	1.71
Missing	1,413	2.10	387	1.31	1,800	2.10
Passive smoking before pregnancy						
No	54,948	81.48	25,212	85.51	80,160	81.48
Occasional	9,485	14.06	3,405	11.55	12,890	14.06
Often	1,367	2.03	460	1.56	1,827	2.03
Missing	1,639	2.43	408	1.38	2,047	2.43
Husbands' smoking in early pregnancy						
No	47,825	70.92	20,440	69.32	68,265	70.92
Decreased	9,960	14.77	4,623	15.68	14,583	14.77
Yes	9,571	14.19	4,356	14.77	13,927	14.19
Missing	83	0.12	66	0.22	149	0.12
parity						
0	48,248	71.54	20,068	68.06	68,316	71.54
≥1	19,191	28.46	9,417	31.94	28,608	28.46

Maternal history of adverse pregnancy outcomes						
No	41,136	61.00	17,425	59.10	58,561	61.00
Yes	26,303	39.00	12,060	40.90	38,363	39.00
Maternal history of chronic diseases						
No	67,019	99.38	29,295	99.36	96,314	99.38
Yes	420	0.62	190	0.64	610	0.62
Maternal birth defects or history of a birth defect in a previous delivery						
No	66,827	99.09	29,245	99.19	96,072	99.09
Yes	612	0.91	240	0.81	852	0.91
**Year**						
**2010**	**7,136**	**10.58**	**232**	**0.79**	**7,368**	**10.58**
**2011**	**6,611**	**9.80**	**935**	**3.17**	**7,546**	**9.80**
2012	12,103	17.95	5,414	18.36	17,517	17.95
2013	14,545	21.57	5,048	17.12	19,593	21.57
2014	12,283	18.21	5,060	17.16	17,343	18.21
2015	12,965	19.22	4,328	14.68	17,293	19.22
**2016**	**1,796**	**2.66**	**8,468**	**28.72**	**10,264**	**2.66**
Areas in Chongqing						
Urban core area	17,295	25.65	4,791	16.25	22,086	25.65
New urban district	31,204	46.27	13,502	45.79	44,706	46.27
Northeast ecological conservation area	14,543	21.56	8,500	28.83	23,043	21.56
Southeast ecological protection area	4,397	6.52	2,692	9.13	7,089	6.52

Response: Yes, pregnant women who were followed up till delivery or pregnancy terminated; No, pregnant women who were lost of follow-up, died or had an ectopic pregnancy.

Table S2. Characteristics of pregnant women according to periconceptional folic acid (FA) supplementation.

Characteristics	Starting at least 3 months before pregnancy (N=21,184)		Starting 1-2 months before pregnancy (N=11,828)		Starting after conception (N=17,392)		No FA supplementation (N=15,239)		Tota[illegible] (N=65,[illegible]
	n	%	n	%	n	%	n	%	n
Maternal age (years)									

20–24	8,437	39.83	5,633	47.62	8,835	50.80	7,730	50.73	30,635
25–29	9,301	**43.91**	4,731	40.00	6,551	37.67	5,229	34.31	25,812
30–34	2,383	**11.25**	1,044	8.83	1,481	8.52	1,495	9.81	6,403
≥35	964	**4.55**	383	3.24	479	2.75	749	4.92	2,575
Missing	99	0.47	37	0.31	46	0.26	36	0.24	218
Maternal ethnicity									
Han	20,273	95.70	11,082	93.69	16,241	93.38	13,449	88.25	61,045
Others	677	3.20	405	3.42	710	4.08	1,034	6.79	2,826
Missing	234	1.10	341	2.88	441	2.54	756	4.96	1,772
Maternal education									
Primary school or below	770	3.63	463	3.91	616	3.54	863	5.66	2,712
Junior high school	7,659	36.15	4,750	40.16	7,404	42.57	7,596	49.85	27,409
Senior high school	4,758	22.46	2,841	24.02	4,599	26.44	3,475	22.80	15,673
College/University or above	7,084	**33.44**	3,074	25.99	4,007	23.04	2,292	15.04	16,457
Missing	913	4.31	700	5.92	766	4.40	1,013	6.65	3,392
Maternal employment									
Farmer	8,149	38.47	4,913	41.54	7,192	41.35	7,758	50.91	28,012
Worker/ waiter	4,202	19.84	2,457	20.77	3,994	22.96	2,861	18.77	13,514
Merchant	750	3.54	406	3.43	614	3.53	491	3.22	2,261
Housewife	1,054	4.98	656	5.55	1,071	6.16	860	5.64	3,641
Teacher/civil servant/staff	4,319	**20.39**	1,830	15.47	2,166	12.45	1,216	7.98	9,531
Others	1,984	9.37	1,108	9.37	1,716	9.87	1,177	7.72	5,985
Missing	726	3.43	458	3.87	639	3.67	876	5.75	2,699
Maternal household registration									
Rural	14,079	66.46	8,746	73.94	13,087	75.25	12,476	81.87	48,388
Urban	7,102	**33.53**	3,081	26.05	4,304	24.75	2,762	18.12	17,249
Missing	3	0.01	1	0.01	1	0.01	1	0.01	6
Passive smoking before pregnancy									
No	16,500	77.89	9,757	82.49	14,402	82.81	12,684	83.23	53,343
Occasional	3,652	**17.24**	1,515	12.81	2,296	13.20	1,858	12.19	9,321
Often	611	**2.88**	247	2.09	302	1.74	190	1.25	1,350
Missing	421	1.99	309	2.61	392	2.25	507	3.33	1,629
Husbands' smoking in early pregnancy									
No	16,801	**79.31**	8,369	70.76	11,020	63.36	10,385	68.15	46,575
Decreased	2,390	11.28	1,901	16.07	3,332	19.16	2,050	13.45	9,673
Yes	1,982	9.36	1,544	13.05	3,013	17.32	2,780	18.24	9,319
Missing	11	0.05	14	0.12	27	0.16	24	0.16	76
Maternal BMI before pregnancy (kg/m^2)									

<18.5	3,240	15.29	1,838	15.54	2,669	15.35	2,078	13.64	9,825
18.5–23.9	15,323	**72.33**	8,453	71.47	12,373	71.14	10,776	70.71	46,925
24–27.9	1,943	9.17	1,089	9.21	1,746	10.04	1,620	10.63	6,398
≥28	328	1.55	190	1.61	256	1.47	317	2.08	1,091
Missing	350	1.65	258	2.18	348	2.00	448	2.94	1,404
Gravidity									
0	9,910	46.78	5,871	49.64	8,480	48.76	6,767	44.41	31,028
≥1	10,909	51.50	5,694	48.14	8,556	49.20	8,015	52.60	33,174
Missing	365	1.72	263	2.22	356	2.05	457	3.00	1,441
Parity									
0	15,752	74.36	8,948	75.65	12,748	73.30	9,843	64.59	47,291
≥1	5,432	25.64	2,880	24.35	4,644	26.70	5,396	35.41	18,352
Maternal history of adverse pregnancy outcomes									
No	12,558	59.28	7,348	62.12	10,748	61.80	9,527	62.52	40,181
Yes	8,626	**40.72**	4,480	37.88	6,644	38.20	5,712	37.48	25,462
Maternal history of chronic diseases									
No	21,008	99.17	11,755	99.38	17,302	99.48	15,174	99.57	65,239
Yes	176	**0.83**	73	0.62	90	0.52	65	0.43	404
Maternal birth defects or history of a birth defect in a previous delivery									
No	20,966	98.97	11,705	98.96	17,267	99.28	15,113	99.17	65,051
Yes	218	**1.03**	123	**1.04**	125	0.72	126	0.83	592
Recruiting Year									
2010	3,476	**16.41**	1,126	9.52	1,268	7.29	1,266	8.31	7,136
2011	2,349	11.09	1,554	13.14	1,500	8.62	1,208	7.93	6,611
2012	3,376	15.94	2,211	18.69	3,142	18.07	3,374	22.14	12,103
2013	4,012	18.94	2,094	17.70	3,902	22.44	4,537	29.77	14,545
2014	3,603	17.01	2,017	17.05	3,547	20.39	3,116	20.45	12,283
2015	4,368	20.62	2,826	23.89	4,033	23.19	1,738	11.40	12,965
Areas in Chongqing									
Urban core area	6,758	**31.90**	2,930	24.77	4,158	23.91	3,124	20.50	16,970
New urban district	10,540	**49.75**	5,661	47.86	7,868	45.24	6,490	42.59	30,559
Northeast ecological conservation area	2,990	14.11	2,599	21.97	4,276	24.59	3,984	26.14	13,849
Southeast ecological protection area	896	4.23	638	5.39	1,090	6.27	1,641	10.77	4,265
Compliance of FA supplementation									
Irregular	831	3.92	841	7.11	1,795	10.32			
regular	20,353	**96.08**	10,987	92.89	15,597	89.68			

Table S3. Risk ratios and 95% CIs for spontaneous abortion according to maternal periconceptional folic acid (FA) supplementation in sensitivity analysis.

	Sensitivity 1 [a]		Sensitivity 2 [b]		Sensitivity 3 [c]	
	aRR	95% CI	aRR*	95% CI	aRR*	95% CI
No supplementation	1.00		1.00		1.00	
Having supplementation	0.51	0.48,0.55	0.51	0.47,0.55	0.49	0.46,0.52
Starting at least 3 months prior to conception	0.45	0.41,0.50	0.45	0.41,0.50	0.43	0.40,0.47
Starting 1-2 months prior to conception	0.55	0.50,0.61	0.54	0.48,0.60	0.52	0.47,0.58
Starting after conception	0.55	0.50,0.59	0.55	0.50,0.60	0.52	0.48,0.57

[a] This sensitivity analyses additionally were adjusted for maternal reproductive tract infection, cytomegalovirus, and toxoplasma infection before pregnancy. [b] This sensitivity analyses were excluded pregnancies of women without information of any covariates. [c] This sensitivity analyses were excluded pregnancies ended with induced abortion/still birth. **Abbreviations:** cRR, crude risk ratio; aRR, adjusted risk ratio; CI, confidence interval.

Table S4. The association between maternal periconceptional folic acid (FA) supplementation and the follow-ups time in gestation.

Maternal periconceptional FA supplementation	Follow-ups time in gestation				χ^2	p
	≤84 days		≥85 days			
	N	%	N	%		
No supplementation	8,107	23.10	7,350	22.73		
Having supplementation	26,982	76.90	24,984	77.27		
Total	35,089		32,334		1.3223	0.250

Table S5. Risk ratios and 95% CIs for spontaneous abortion (SA) according to maternal periconceptional folic acid (FA) supplementation in a subpopulation recruited in 2010 (N=7 136).

Maternal periconceptional FA supplementation	No. of Pregnancies	SAs, No. (%)	Model 1		Model 2 [a]	
			cRR	95% CI	aRR	95% CI
No supplementation	1,266	52 (4.11)	1.00	-	1.00	-
Having supplementation	5,870	51 (0.87)	0.21	0.14,0.31	0.27	0.18,0.41
Starting at least 3 months prior to conception	3,476	18 (0.52)	0.13	0.07,0.21	0.19	0.11,0.34 [b]
Starting 1-2 months prior to conception	1,126	9 (0.80)	0.19	0.10,0.39	0.24	0.12,0.47 [b]
Starting after conception	1,268	24 (1.89)	0.46	0.29,0.74	0.41	0.25,0.67 [b]

[a] aRR adjusted for maternal BMI, passive smoking before pregnancy, history of adverse pregnancy outcomes, chronic diseases, birth defects or history of a birth defect in a previous delivery, and husbands' smoking in early pregnancy, in addition to maternal age, ethnicity, education, employment, household registration, and area of recruitment. [b] aRR for FA supplementation initiated at least 3months prior to conception was significantly lower than FA supplementation after conception (χ^2=5.10, p=0.0239). **Abbreviations:** cRR, crude risk ratio; aRR, adjusted risk ratio; CI, confidence interval.

Table S6. Risk ratios and 95% CIs for pregnancies ending with still birth or induced abortion due to foetal abnormalities according to maternal periconceptional folic acid (FA).

Maternal periconceptional FA supplementation	No. of Pregnancies	Induced abortions or still births, No. (%)	Model 1		Model 2 [a]	
			cRR	95% CI	aRR	95% CI
No supplementation	13,878	1,028 (7.40)	1.00	-	1.00	-
Having supplementation	48,241	1,049 (2.17)	0.29	0.27,0.32	0.31	0.29,0.34
Starting at least 3 months prior to conception	20,383	388 (1.90)	0.26	0.23,0.29	0.28	0.25,0.32 [b]
Starting 1-2 months prior to conception	11,287	231 (2.05)	0.28	0.24,0.32	0.29	0.25,0.34 [b]
Starting after conception	16,571	430 (2.59)	0.35	0.31,0.39	0.35	0.31,0.39 [b]

[a] aRR adjusted for maternal BMI, passive smoking before pregnancy, history of adverse pregnancy outcomes, chronic diseases, birth defects or history of a birth defect in a previous delivery, and husbands' smoking in early pregnancy, in addition to maternal age, ethnicity, education, employment, household registration, year and area of recruitment. [b] aRR for FA supplementation initiated 3 months or more (χ^2=8.60, p=0.0034), or initiated 1-2 months (χ^2=4.62, p=0.0315) prior to conception was significantly lower than supplementation after conception. **Abbreviations:** cRR, crude risk ratio; aRR, adjusted risk ratio; CI, confidence interval
